# Congenitally corrected transposition of the great arteries and coronary artery disease: a case report in an 83-year-old male

**DOI:** 10.1093/ehjcr/ytaf161

**Published:** 2025-05-07

**Authors:** Ahmed Alomrani, Mohammed Alshammari, Fahad Bindakhil, Hadi Shabi, Abdullah Alkhodair

**Affiliations:** Department of Cardiac Sciences, Ministry of National Guard Health Affairs, PO Box 22490, Riyadh 11426, Saudi Arabia; College of Medicine, King Saud bin Abdulaziz University for Health Sciences, PO Box 3660, Riyadh 11481, Saudi Arabia; College of Medicine, King Saud bin Abdulaziz University for Health Sciences, PO Box 3660, Riyadh 11481, Saudi Arabia; College of Medicine, King Saud bin Abdulaziz University for Health Sciences, PO Box 3660, Riyadh 11481, Saudi Arabia; King Fahad Medical City, PO Box 59046, Riyadh 11525, Riyadh Region, Saudi Arabia

**Keywords:** Congenitally corrected transposition of the great arteries (CCTGA), Coronary artery disease (CAD), Case report, Percutaneous coronary intervention (PCI)

## Abstract

**Background:**

Congenitally corrected transposition of the great arteries (CCTGA), or L-loop TGA, is a rare congenital heart defect, comprising <1% of congenital heart diseases, with an incidence of ∼1 in 33 000 births. It is characterized by atrioventricular and ventriculo-arterial discordance, where the left ventricle connects to a right atrium and pumps deoxygenated blood into the pulmonary artery, while the right ventricle (RV) connects to a left atrium and pumps oxygenated blood into the aorta. Congenitally corrected transposition of the great arteries often coexists with other cardiac anomalies, although ∼10% of cases are isolated. A case report highlights a unique instance of coronary artery disease (CAD) in CCTGA.

**Case summary:**

An 83-year-old male with CCTGA presented with persistent nausea, fatigue, poor oral intake, and epigastric pain. He had a history of hypertension, Type II diabetes, dyslipidaemia, ischaemic heart disease, and chronic atrial fibrillation. On examination, he was stable but showed signs of a urinary tract infection. A cardiac workup revealed no ischaemic changes on electrocardiography, but a cardiac computed tomography identified significant CAD involving multiple vessels. A decision was made to perform percutaneous coronary intervention on the right coronary artery, successfully placing two stents.

**Discussion:**

In patients with CCTGA, major factors contributing to morbidity and mortality include progressive decline in systemic RV function and systemic tricuspid valve regurgitation. A retrospective study showed that 25% of uncomplicated CCTGA patients develop heart failure by age 45, while approximately two-thirds of complicated cases do. Survival beyond age 70 is rare. Prompt management of CAD through angioplasty is critical to prevent further deterioration of RV function and worsening tricuspid regurgitation. However, the atypical positioning of the aorta and coronary arteries complicates selective coronary angiography, making a challenging diagnosis and treatment.

**Learning points**

- Atypical presentation of coronary artery disease (CAD): Patients with congenitally corrected transposition of the great arteries (CCTGA) may present with atypical symptoms of CAD, such as non-specific epigastric pain or fatigue. This warrants a high index of suspicion for cardiac ischaemia, even in the absence of typical chest pain.
- Prioritize systemic right ventricle in CAD management: In CCTGA, the systemic right ventricle is more vulnerable to ischaemic damage, making early and targeted coronary interventions crucial to avoid severe right ventricular dysfunction and worsening of atrioventricular valve regurgitation.

## Introduction

Congenitally corrected transposition of the great arteries (CCTGA), also known as L-loop TGA, is a rare congenital heart defect, accounting for <1% of all congenital heart diseases.^1^ It has an incidence of around 1:33 000 births.^2^ Congenitally corrected transposition of the great arteries is characterized by atrioventricular discordance and ventriculo-arterial discordance. The left ventricle is connected to a normally positioned right atrium, from which it receives deoxygenated blood through the mitral valve and pumps it directly into the pulmonary artery. On the left side, the right ventricle (RV) is connected to the left atrium (LA) and pumps oxygenated blood received through the tricuspid valve into the aorta. Despite the morphological RV functioning as the systemic ventricle, a physiologically normal blood flow is achieved due to the double discordance.

However, this ventricular discordance places the RV and tricuspid valve under systemic pressure, often leading to a gradual decline in RV function.^2^ Moreover, CCTGA frequently presents alongside additional cardiac anomalies like ventricular septal defects and pulmonary artery stenosis.^3^ However, ∼10% of CCTGA cases present isolated and uncomplicated by any other anomaly.^3^

Notable anatomical variations in coronary arteries have been documented in the literature.^4^ A left-dominant coronary circulation was reported, with the left circumflex artery (LCX) and intermediate artery arising separately from the left sinus and the left anterior descending artery (LAD) originating from the proximal right coronary artery (RCA) via a single ostium from the right coronary sinus.^4^ In such cases, revascularization for coronary artery disease (CAD) can pose considerable challenges. Given the rarity of CCTGA, there is limited literature discussing the management of CAD in this population. Therefore, this case report presents a rare occurrence of CAD in an 83-year-old male with CCTGA.

## Summary figure

**Table:** 

1941	Congenitally corrected transposition of the great arteries (CCTGA) diagnosed at birth
1999	History of ischaemic heart disease with significant lesion in the left coronary system
March 2023	Electrocardiogram revealing atrial fibrillation
July 2023	Hospital admission for workup of cardiac ischaemia due to suspicion of atypical presentation of acute coronary syndrome Cardiac CT and coronary angiogram showing moderate to severe coronary artery disease Multidisciplinary team discussion and targeted percutaneous coronary intervention to the right coronary artery Discharge
August 2023	Follow-up visit

## Case presentation

An 83-year-old male previously diagnosed with CCTGA, uncomplicated by any cardiac defect or valvular lesion, presented to the clinic with persistent nausea, fatigue, poor oral intake, and non-specific epigastric pain for 2 weeks.

The patient is a known case of hypertension, Type II diabetes mellitus, dyslipidaemia, and benign prostatic hypertrophy, all of which are controlled with medications. Additionally, he has non-cardiac cirrhosis, partial adrenal insufficiency, a history of ischaemic heart disease. The patient has chronic atrial fibrillation controlled with beta-blockers and is on anticoagulation with apixaban 2.5 mg BID. The CHA₂DS₂-VASc score of 5 indicates a high stroke risk, while the Charlson Comorbidity Index score of 6 reflects significant comorbidities. The HAS-BLED score of 2 suggests a moderate risk for major bleeding.

Upon admission, the patient was afebrile, alert, oriented, and vitally stable with a blood pressure of 161/71 mmHg, pulse rate of 79 b.p.m., and oxygen saturation of 99% on room air. Physical examination revealed an irregular rhythm consistent with atrial fibrillation, without an appreciable heart murmur. The chest was clear, with no added sounds or crackles, and the abdomen was soft and lax with tenderness in the lower abdomen. Urinalysis was positive and suggestive of a urinary tract infection. The patient was started on empiric intravenous antibiotic therapy, with targeted antibiotics administered based on culture results. Despite treatment, the patient continued to experience persistent abdominal pain and shortness of breath on exertion.

**Figure 1:**
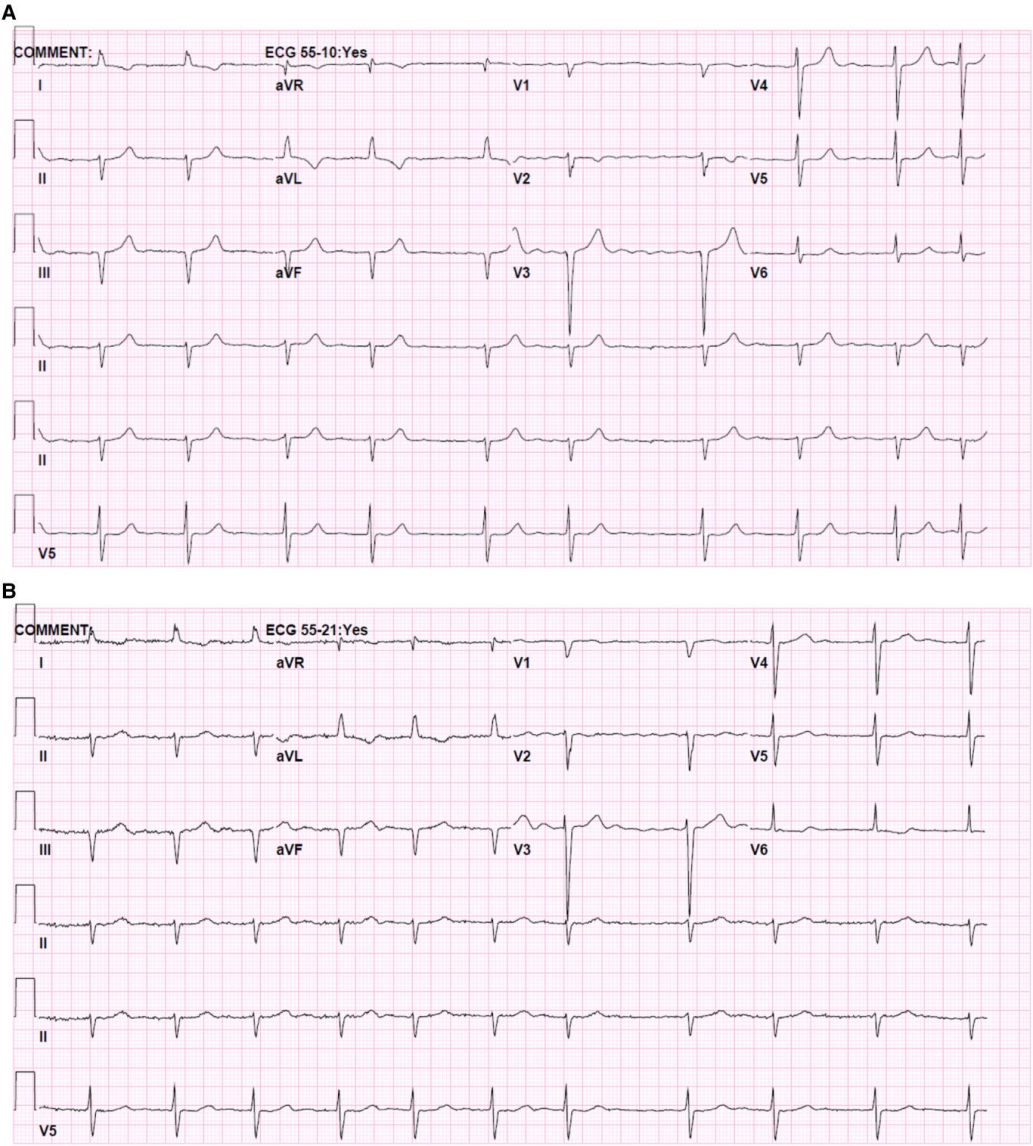
Electrocardiography showing atrial fibrillation (*A*) before and (*B*) after percutaneous coronary intervention.

**Figure 2:**
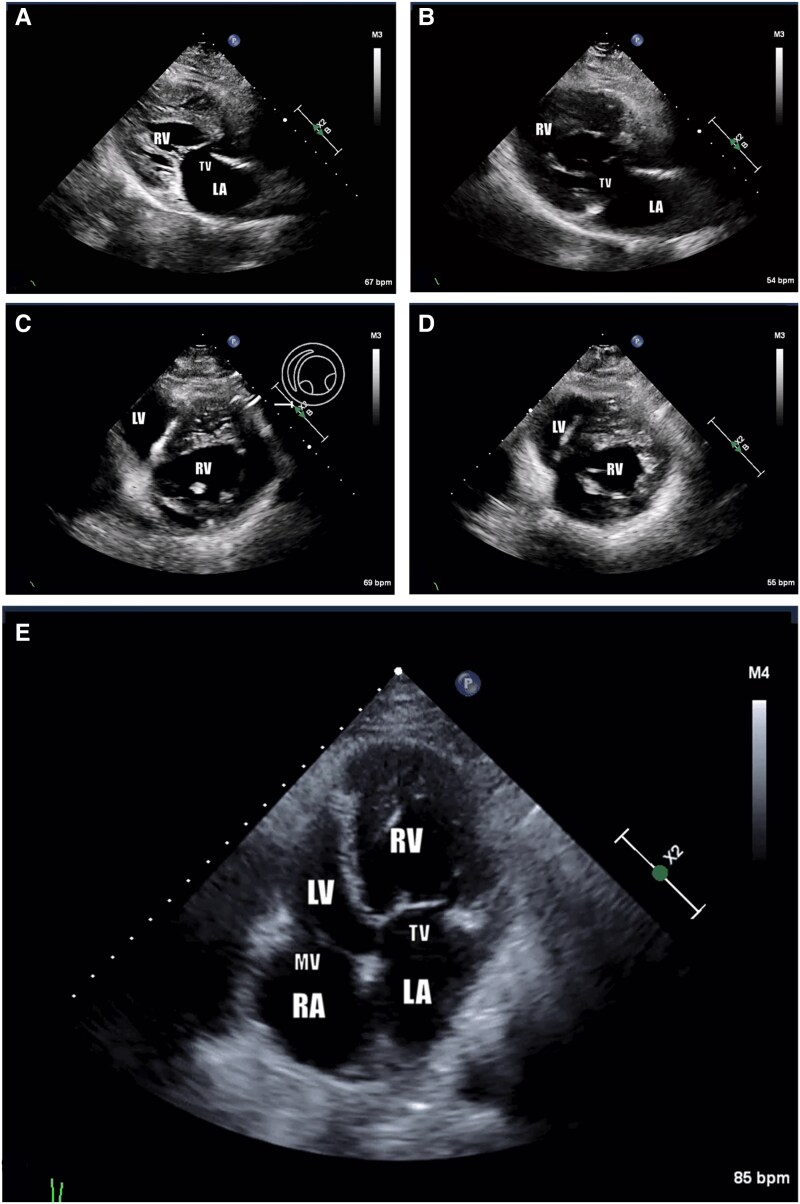
Echocardiogram findings: consistent with congenitally corrected transposition of the great arteries (levo-transposition of the great arteries). Systemic ventricle (morphologically right ventricle) is receiving blood via tricuspid valve and pump it through the aortic valve, which is located anterior to the PV. Systemic ventricle is mildly dilated and heavily trabeculated with mild to moderate global hypokinesis and mildly reduced systolic function (ejection fraction 45%–50%). There is mild tricuspid regurgitation. (*A*) Pre-catheterization parasternal long-axis view. (*B*) Post-catheterization parasternal long-axis view. (*C*) Pre-catheterization short-axis mid-ventricular view. (*D*) Post-catheterization short-axis mid-ventricular view. (*E*) Post-catheterization apical four-chamber view. Note: AFib. AV, aortic valve; LA, left atrium; LV, left ventricle; MV, mitral valve; RA, right atrium; RV, right ventricle; TV, tricuspid valve.

Given the suspicion of an atypical presentation of acute coronary syndrome (ACS), the patient was admitted for cardiac ischaemia workup. Electrocardiography showed atrial fibrillation without signs of ischaemia, and troponin levels were normal (*Figure 1*). However, transthoracic echocardiography demonstrated mildly reduced ejection fraction (heart failure with midrange ejection fraction 45–50%) in the systemic ventricle (morphological RV) and mild tricuspid regurgitation (*Figure 2*). Given the suspected atypical presentation of ACS, negative troponin levels, and depressed systemic ventricular function, a cardiac computed tomography (CT) scan was ordered, confirming CCTGA anatomy and revealing significant CAD involving multiple coronary arteries.

**Figure 3:**
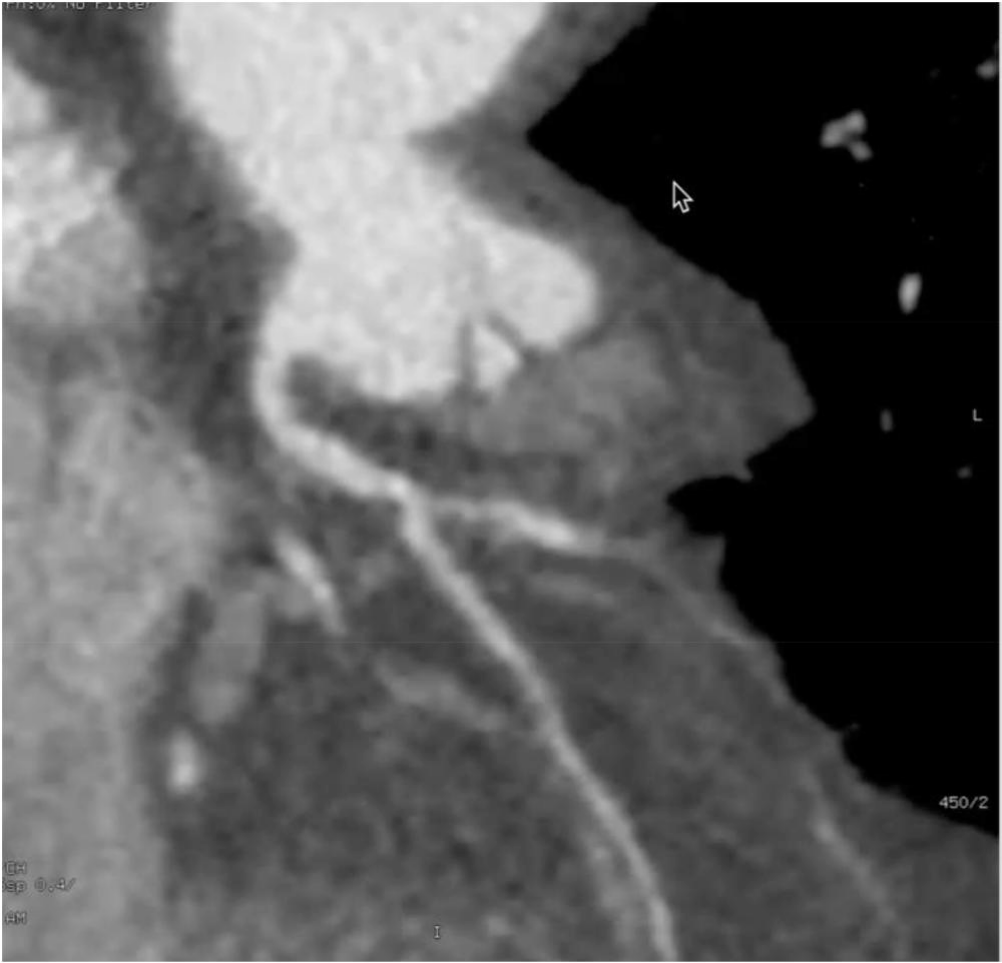
Cardiac computed tomography findings: the right facing cusp gives rise right coronary artery; the proximal segment prior to its bifurcation has moderate to severe coronary artery disease. Coronary artery calcium score is 100.

In the cardiac CT, two major coronary arteries were identified, one arising from the left-facing cusp and the other from the right-facing cusp. The right-facing cusp gave rise to a non-dominant RCA and part of the LAD. The proximal LAD segment, prior to bifurcation, showed moderate to severe CAD (50%–75% stenosis), while the RCA and LAD beyond this segment showed no significant disease. The left-facing cusp gave rise to a large, dominant LCX with its branches and one diagonal branch. The LCX showed mild CAD in the proximal segment and severe CAD in the distal segment (>75% stenosis). The diagonal branch and obtuse marginal artery exhibited moderate diffuse CAD in their proximal segments. The coronary artery calcium score was 100, indicating the presence of mild coronary artery calcification (*Figure 3*).

**Figure 4:**
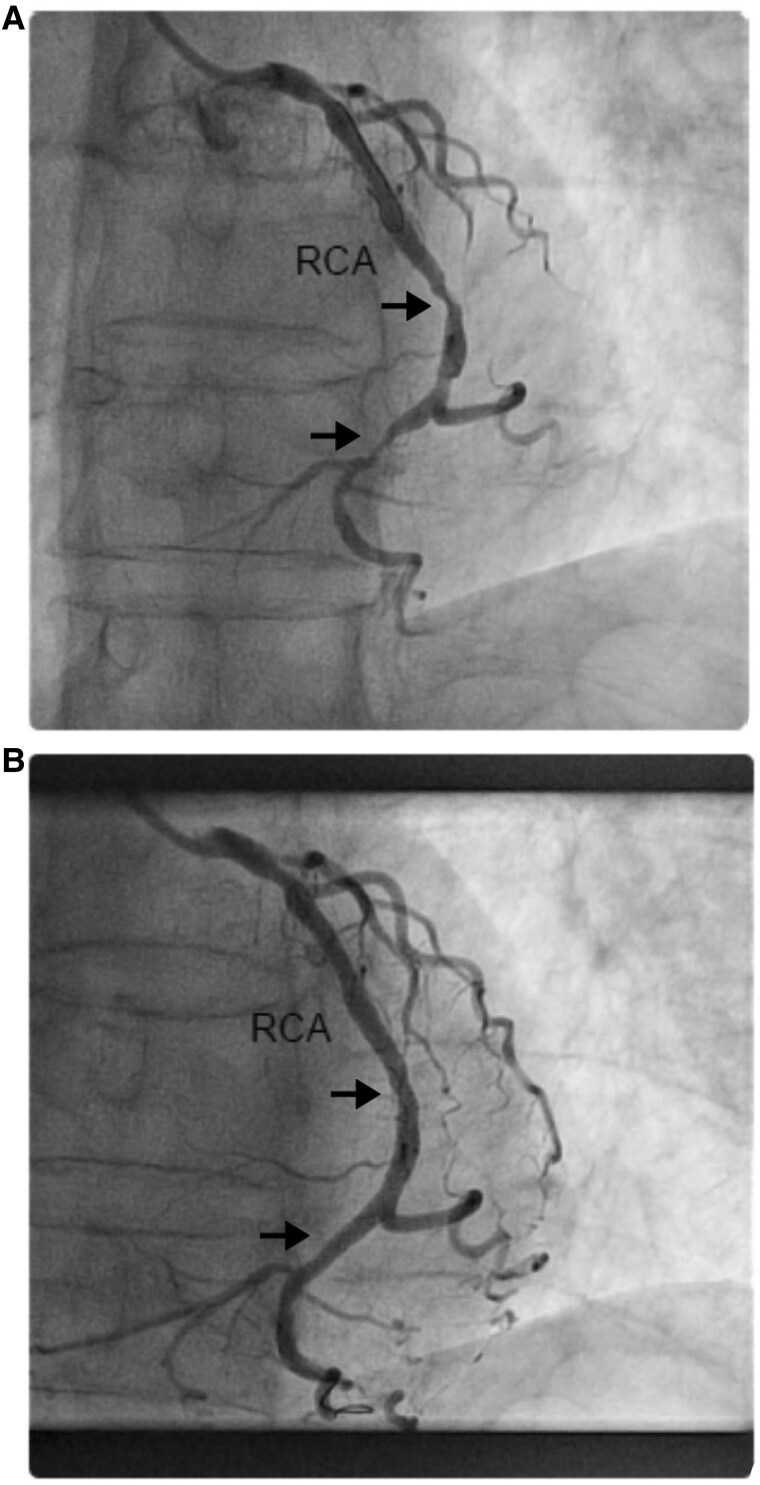
Coronary angiogram: (*A*) Right anterior oblique projection with two significant stenoses, solid arrow heads. (*B*) Right anterior oblique projection, post-percutaneous coronary intervention, resolved stenosis, solid arrow heads. RCA, right coronary artery.

Subsequent diagnostic coronary angiogram revealed mild stenosis of the left main artery, severe ostial and proximal stenosis (80%) of the LAD, severe stenosis (90%) of the LCX, and proximal severe stenosis (90%) of the RCA. It was noted that the systemic ventricle (morphological RV) depended on the supply from the RCA (*Figure 4*).

A multidisciplinary team collectively recommended a targeted percutaneous coronary intervention (PCI) for the RCA, given its role in supplying the systemic ventricle. Two stents were successfully placed in the mid and distal RCA without complications. The initial outcome was favourable, and the patient's symptoms improved. At discharge, the patient prescribed clopidogrel 75 mg q24hr, atorvastatin 40 mg q24hr, lisinopril 5 mg q24hr, bisoprolol 1.25 mg q24hr, apixaban 2.5 mg BID, tamsulosin 0.4 mg q24hr, and esomeprazole 40 mg q24hr.

At 6-week follow-up visit, the patient is asymptomatic at low level activity consistent with New York Heart Association (NYHA) Class II with no exertional chest pain. Echocardiography showed improved systemic ventricular function and mild tricuspid regurgitation (*Figure 2*). Subsequent follow-up was unremarkable.

## Discussion

The primary pathologies affecting morbidity and mortality in CCTGA patients include the progressive decline in systemic RV function and regurgitation of the systemic tricuspid valve. Progressive regurgitation of the tricuspid valve is the most frequent association, occurring in 90% of cases.^5^ A retrospective study reviewing 182 CCTGA patients revealed that heart failure developed by age 45 in 25% of uncomplicated CCTGA cases, compared with around two-thirds of complicated cases.^6^ As a result, there are only a few reported cases of survival beyond the age of 70 years.^5^

Patients with uncomplicated CCTGA primarily experience a progressive decline in RV function, which may be their sole symptom, and may develop diseases commonly seen in the general population, such as hypertension, diabetes mellitus, and other cardiovascular risk factors, predisposing them to CAD.^7^

In a CCTGA patient, myocardial infarction caused by occlusion of the proximal RCA could further impair the already compromised systemic RV, potentially leading to catastrophic consequences. Early management of CAD through angioplasty is therefore essential to prevent deterioration of systemic RV function and worsening of tricuspid valve regurgitation. However, the anatomical configuration complicates selective coronary angiography, as the RCA contributes to part of the LAD and supplies the systemic RV.

The RCA showed significant angiographic disease in our patient with regional wall abnormalities and systemic ventricular dysfunction suggestive of ischaemia. Given the patient’s age, comorbidities, and frailty, along with the lack of published physiological studies in this anatomy, our team collectively recommends targeted PCI to the RCA, which supplies the systemic ventricle.

Reports on CAD in CCTGA patients are limited in the literature. Furthermore, these patients may present with complex coronary anatomy or concurrent cardiac anomalies, complicating their management.^3^ Therefore, additional reports are necessary to enhance understanding and develop appropriate approaches for this rare condition.

## Conclusion

This is a case of an 83-year-old male with multiple cardiovascular risk factors who developed severe CAD affecting the arteries supplying the systemic ventricle (morphological RV), which already exhibited a mild reduction in ejection fraction. Targeted PCI was successfully performed, yielding favourable outcomes.

In conclusion, this case highlights the challenges faced by patients with CCTGA who develop CAD. Given the scarcity of literature addressing CAD in CCTGA patients, each case presents a unique clinical scenario that demands a multidisciplinary approach for optimal management.

This report emphasizes the importance of early recognition and intervention in patients with CCTGA presenting with symptoms suggestive of CAD. Timely intervention can prevent further deterioration of RV function and tricuspid valve regurgitation, thereby improving long-term outcomes.

Moving forward, accumulating additional case reports and studies is essential to enhance our understanding of CAD in CCTGA patients and refine management strategies.

## Supplementary Material

ytaf161_Supplementary_Data

## Data Availability

The information in this article was gathered from our institution's database with the patient's consent. The data can be shared upon request to the authors, provided the patient agrees.

## Lead author biography

Ahmed Alomrani is a paediatric and adult interventional cardiologist and pulmonary hypertention.

## Notes

**Consent:** The patient has provided consent to publish this case report in accordance with COPE guidelines.

**Funding:** No funding was required.
